# Metagenome Analysis of a Complex Community Reveals the Metabolic Blueprint of Anammox Bacterium “*Candidatus* Jettenia asiatica”

**DOI:** 10.3389/fmicb.2012.00366

**Published:** 2012-10-29

**Authors:** Ziye Hu, D. R. Speth, Kees-Jan Francoijs, Zhe-Xue Quan, M. S. M. Jetten

**Affiliations:** ^1^Department of Microbiology, Institute for Water and Wetland Research, Radboud University NijmegenNijmegen, Netherlands; ^2^Department of Molecular Biology NCMLS, Radboud University NijmegenNijmegen, Netherlands; ^3^Department of Microbiology and Microbial Engineering, School of Life Sciences, Fudan UniversityShanghai, China; ^4^Department of Biotechnology, Delft University of TechnologyDelft, Netherlands

**Keywords:** “*Ca* Jettenia asiatica”, metagenome, complex community

## Abstract

Anaerobic ammonium-oxidizing (anammox) bacteria are key players in the global nitrogen cycle and responsible for significant global nitrogen loss. Moreover, the anammox process is widely implemented for nitrogen removal from wastewaters as a cost-effective and environment-friendly alternative to conventional nitrification-denitrification systems. Currently, five genera of anammox bacteria have been identified, together forming a deep-branching order in the Planctomycetes-Verrucomicrobium-Chlamydiae superphylum. Members of all genera have been detected in wastewater treatment plants and have been enriched in lab-scale bioreactors, but genome information is not yet available for all genera. Here we report the metagenomic analysis of a granular sludge anammox reactor dominated (∼50%) by “*Candidatus* Jettenia asiatica.” The metagenome was sequenced using both Illumina and 454 pyrosequencing. After *de novo* assembly 37,432 contigs with an average length of 571 nt were obtained. The contigs were then analyzed by BLASTx searches against the protein sequences of “*Candidatus* Kuenenia stuttgartiensis” and a set of 25 genes essential in anammox metabolism were detected. Additionally all reads were mapped to the genome of an anammox strain KSU-1 and *de novo* assembly was performed again using the reads that could be mapped on KSU-1. Using this approach, a gene encoding copper-containing nitrite reductase NirK was identified in the genome, instead of cytochrome *cd*_1_-type nitrite reductase (NirS, present in “*Ca*. Kuenenia stuttgartiensis” and “*Ca*. Scalindua profunda”). Finally, the community composition was investigated through MetaCluster analysis, 16S rRNA gene analysis and read mapping, which showed the presence of other important community members such as aerobic ammonia-oxidizing bacteria, methanogens, and the denitrifying methanotroph “*Ca*. Methylomirabilis oxyfera”, indicating a possible active methane and nitrogen cycle in the bioreactor under the prevailing operational conditions.

## Introduction

Two microbial processes are responsible for the release of fixed nitrogen: denitrification and anaerobic ammonium oxidation (anammox). Anammox bacteria oxidize ammonia to dinitrogen gas under anaerobic conditions with nitrite as the electron acceptor. The anammox process is widely applied for wastewater treatment and has economical and environmental advantages over the conventional nitrogen removal processes nitrification and denitrification (Jetten et al., 1997; Van Dongen et al., 2001; Siegrist et al., 2008; Kartal et al., 2010). Furthermore, anammox bacteria are important players in the global nitrogen cycle, and widely distributed in various ecosystems (citations). It is now estimated that they contribute significantly to global nitrogen loss (Thamdrup and Dalsgaard, 2002; Kuypers et al., 2005; Hamersley et al., 2007; Humbert et al., 2010).

**Figure 1:**
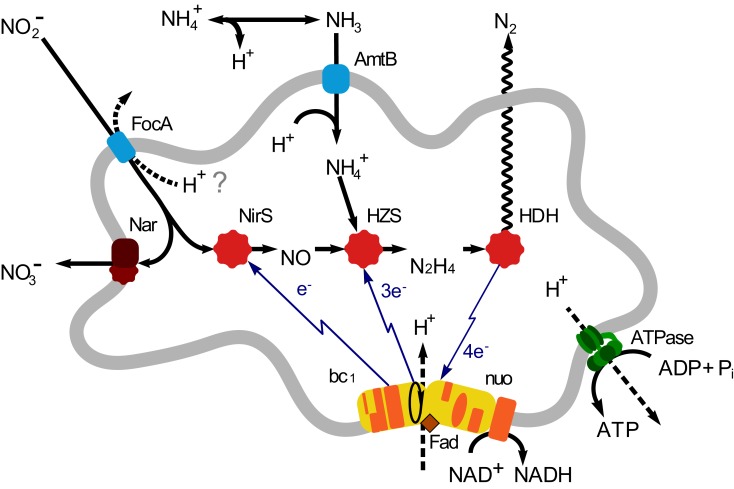
**Overview of anammox metabolism in “*Candidatus* Jettenia asiatica”**. AmtB, ammonium transport protein; FocA, Nitrite transporter; nitrite/formate transporter; NirS, Nitrite reductase; Nar, Nitrate reductase; HZS, Hydrazine synthesis; HDH, hydrazine dehydrogenase; bc_1_, cytochrome *bc*_1_ complex; Nuo, NADH ubiquinone oxidoreductase.

Until now five genera of anammox bacteria have been identified: “*Candidatus* Brocadia”, “*Candidatus* Kuenenia”, “*Candidatus* Scalindua”, “*Candidatus* Anammoxoglobus”, and “*Candidatus* Jettenia”, together forming a monophyletic order *Brocadiales* that ranches deeply in the phylum *Planctomycetes* (Jetten et al., 2010). Various aspects of the anammox bacteria, such as growth and metabolism, biochemistry and bio-energetics, cell biology, application, and environmental importance, have been addressed in various studies (reviewed in Jetten et al., 2009; van Niftrik and Jetten, 2012). Using environmental shotgun sequencing, which is used to retrieve microbial genomes or genomic fragments from complicated environmental samples, the first metagenome of an anammox bacterium was sequenced from an enrichment culture (∼75%) of “*Candidatus* Kuenenia stuttgartiensis” (hereafter: *Kuenenia*; Strous et al., 2006). Based on this metagenome, the central metabolic pathways of anammox bacteria were predicted and resolved (Strous et al., 2006; de Almeida et al., 2011; Kartal et al., 2011). Based on these findings, the anammox catabolism may be divided into three steps: the reduction of nitrite to NO catalyzed by a *cd*_1_ nitrite: nitric oxide oxidoreductase (NirS); the conversion of equimolar amounts of NO, and ammonium to hydrazine catalyzed by hydrazine synthase (HZS), and finally the oxidation of hydrazine to dinitrogen gas catalyzed by hydrazine dehydrogenase (HDH; de Almeida et al., 2011; Figure 1).

However, the metabolic processes described above and the proteins catalyzing these were predicted based on the genome of *Kuenenia*. Recently, the genome sequences of three more anammox species were reported; a marine species “*Candidatus* Scalindua profunda” (hereafter: *Scalindua*), a waste water species KSU-1, and a freshwater species “*Candidatus* Brocadia fulgida” (Hereafter: *Brocadia*) and all of them showed differences to *Kuenenia* (Gori et al., 2011; Hira et al., 2012; Van de Vossenberg et al., 2012). This indicated that genomic analyses of other anammox bacteria could contribute to a more comprehensive understanding of the metabolic features of these extraordinary microorganisms. To this end, a metagenome analysis was performed on an anammox enrichment dominated by *Candidatus* “Jettenia asiatica” (hereafter: *Jettenia*). Important anammox genes in the dataset were identified and compared to those in the genomes of *Kuenenia* and the anammox strain KSU-1 (Hira et al., 2012).

## Materials and Methods

### Metagenomic sequencing and assembly

Bulk community DNA was extracted from the granular sludge of an anaerobic bioreactor. The sludge was dominated by *Jettenia* (∼50%) as determined by 16S rRNA gene quantitative PCR assays (Quan et al., 2008). The community DNA was isolated and sequenced using Illumina and 454 pyrosesquencing, generating 16,296,896 reads with an average length of 33.6 nt and 162,543 reads with an average length of 215 nt respectively. *De novo* assembly was performed with the CLC genomics workbench (v. 5.1; CLCbio) using default setting (Word size: 22; Bubble size: 50; Minimum contig length: 200; Insertion: 3; Deletion cost: 2). After assembly, ∼33% of the reads were assembled into 37,432 contigs with an average length of 570 nt, about 1/3 of the contigs were longer than 500 nt and 2,549 contigs were longer than 1000 nt, the longest being 4.3 kb.

**Figure 2:**
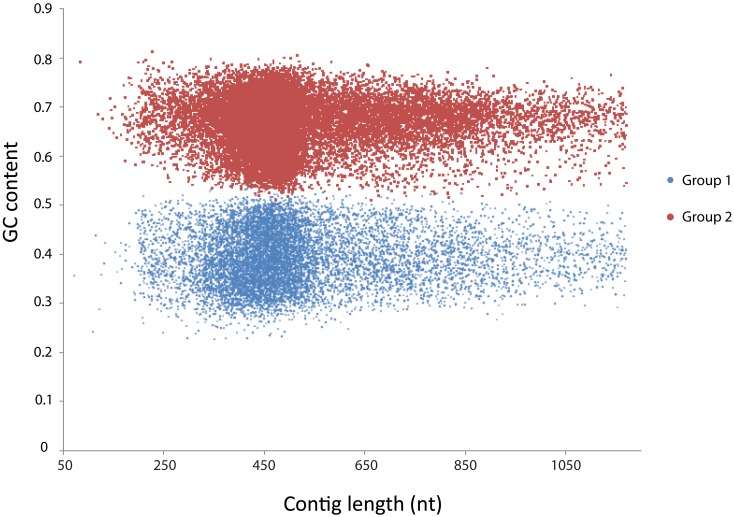
**MetaCluster binning of the *de novo* assembly yielded two bins, clearly separated by GC content**. Group 1 (blue) consisted of 10,343 contigs with an average GC content of 39.4%. Group 2 (red) consisted of 22,939 contigs with an average GC content of 66.2%.

The contigs had a highly diverse GC content and coverage and many of them probably belonged to other community members rather than *Jettenia*. To select contigs which most likely originate from *Jettenia*, two approaches were used:

First, all contigs were used as query in a BLASTx search against the 4,663 proteins of *Kuenenia* using a cutoff of *E*-value < 1^−10^. After that, 12,850 contigs that had hits with *Kuenenia* proteins were retrieved and used as query in a new BLASTx search against NCBI protein database (nr database). Only contigs with best hits with *Kuenenia* and BLAST *E*-value < 1^−10^ and hit length >50 were considered as contigs highly likely to belong to *Jettenia*. A total of 1,187 contigs were obtained in this way. Although the remaining contigs did not have a best hit with *Kuenenia*, 150 contigs did not have any hit within the nr database and 2062 contigs have best hits with *Kuenenia* with *E*-value > 0.001 and 5779 between 0.001 and 1*E*^−10^. For these contigs it was difficult to decide if they belong to *Jetteni*a. Therefore the following approach was also used: All 37,432 contigs were binned using the MetaCluster 3.0 software (Wang et al., 2012). The binning separated the contigs into two groups, containing 10,343 and 22,929 contigs, respectively. The GC content of the two groups was clearly different (Figure 2).

Contigs belonging to group 1 had an average GC content of 39.4%, which was much lower than contigs of group 2 (66.9%). The average length and coverage of contigs in group 1 were higher than contigs belonging to group 2, suggesting that these contigs belonged to the dominant organism in the enrichment. Also, previously sequenced anammox bacteria all had GC contents close to 40% (Strous et al., 2006; Gori et al., 2011; Van de Vossenberg et al., 2012).

In addition, 28 important anammox genes involved in nitrogen metabolism and two genes encoding acetyl-CoA synthase of *Kuenenia* were selected as a reference protein dataset and gene products of these were downloaded from NCBI database. All 37,432 contigs were used as queries in a BLASTx search against this reference protein dataset with a cutoff *E*-value < 1^−10^. Contigs with best hits with the reference protein dataset were retrieved and used as queries again in a new BLASTx search against the NCBI nr database to ensure that they still were the best hit with the target *Kuenenia* proteins (Table 1). All contigs listed in Table 1, except contig270, contig5773, and contig10239, belonged to group 1 (10,343 contigs), which further strengthened the notion that contigs within this group were likely to belong to *Jettenia*.

**Table 1:** **Important anammox genes mapped by *Jettenia* contigs (First assembly)**.

Assumed function	Gene	Gene identification	Protein length (aa)	Jettenia contig	Length contig	Coverage contig	*E*-value	Identity (%)	Mapped length (aa)	Percentage covered
Nitrate/nitrite	nark	kuste2335	405	2336	1598	17.9	9.00E−149	81	391	96.5%
antiporter	nark	kuste2308	389	12331	413	3.3	7.00E−50	70	121	31.1%
Nitrite transporter	focA	kusta0004	363	18259	439	21.1	2.00E−73	86	145	39.9%
	focA	kusta0009	304	3748	420	23.5	4E−66	73	139	45.7%
	focA	kuste3055	300	4712	600	18.5	6.00E−93	78	201	67.0%
	focA	kustd1720	296	ND						
	focA	kustd1721	303	ND						
	focA	kuste4324	304	ND						
Nitrite reductase	nirS	kuste4136	621	ND						
NO forming	nirK		337	1180	872	12.6	6.00E−100	91	195	93.8%
				21694	495	18.5	4.00E−67	93	121	
Ammonium	amtB	kustc0381	585	10957	776	17.9	5.00E−102	88	227	38.8%
transport protein	amtB	kustc1015	448	4905	1723	16.9	1.00E−54	53	181	40.4%
	amtB	kuste3690	679	851	3084	22.1	5.00E−106	48	455	67.0%
				12990	889	18.2	8.00E−103	71	119	
	amtB	kustc1012	451	27696	1147	16.7	9.00E−117	76	245	98.7%
				1921	1396	18.2	1.00E−64	73	157	
	amtB	kustc1009	519	1692	957	16.8	7.00E−59	50	248	47.8%
Hydrazine or	hao	kusta0043	591	32273	680	18.1	4.00E−115	89	225	38.1%
hydroxylamine	hao	kustc0458	554	12792	2044	23.0	0	84	513	92.6%
Oxidation	hzo	kustc0694	656	8275	2004	37.0	0	88	559	85.2%
	hzo	kustd1340	577	8275	2004	37	0	89	551	95.5%
	hao	kustc1061	536	6703	792	23.8	7.00E−144	85	262	48.9%
	hao	kuste2435	499	3813	1075	19.3	5.00E−149	69	328	65.7%
	hao	kuste2457	433	24832	519	19.4	2.00E−20	38	112	25.9%
	hao	kuste4574	584	4414	2406	18.0	2.00E−111	80	242	71.6%
				18139	607	18.9	1.00E−112	88	176	
Hydrazine	hzsA	kuste2861	809	297	3125	51.9	2.00E−163	83	314	99.9%
synthesis				270	2775	30.1	0	81	494	
	hzsB	kuste2860	353	297	3125	51.9	0	84	346	98.0%
	hzsC	kuste2859	386	297	3125	51.9	1.00E−158	80	337	87.3%
Nitrate reductase	narG	kustd1700	1148	1800	2043	41.4	0	84	632	55.1%
				5773	1170	9.8	3.00E−92	55	288	
	narH	kustd1703	410	3327	1262	18.7	1.00E−172	75	375	91.5%
CO_2_ fixation	acsA	kustd1546	653	29038	453	4.9	2.00E−51	78	151	48.5%
				29875	557	19.8	2.00E−74	90	166	
	acsB	kustd1545	727	11325	965	17.1	2.00E−108	93	195	86.7%
				1037	478	15.7	2.00E−83	92	157	
				8268	851	17.0	7.00E−138	85	276	
				10239	385	10.2	3.00E−51	86	88	
				10240	399	5.8	1.00E−57	93	133	

### Mapping reads to KSU-1 genome

Previous phylogenetic analysis based on 16S rRNA genes indicated that *Jettenia* was most closely related (98% similarity of 16S rRNA gene) to an anammox bacterium strain called KSU-1 (Quan et al., 2008; Viancelli et al., 2011). Additionally, the HZS subunit A (*hzsA*), a phylomarker for anammox bacteria, of *Jettenia* and KSU-1 shared 92.8% sequence identity on nucleotide level and 97.0% on amino acid level, which was much higher than any other pair of anammox bacteria (Harhangi et al., 2012). Therefore, in order to achieve a more efficient assembly, four large genome contigs of anammox strain KSU-1 were obtained from NCBI database (GenBank accession number: NZ_BAFH01000001-NZ_BAFH01000004) as reference sequences. All reads generated by both sequencing methods were then mapped to the reference sequences. In total, 76% of the reference sequences were mapped by 1,060,817 reads. The reads were then extracted and used for a new *de novo* assembly, which generated 3,209 contigs with an average length of 840 nt. The BLASTx search of *Jettenia* contigs against the Kuenenia reference protein dataset was performed again now using the 3,209 contigs generated by the new assembly as query (Table 2).

**Table 2:** **Important anammox genes mapped by *Jettenia* contigs (Second assembly)**.

Assumed function	Gene	Gene identification	Protein length (aa)	Jettenia contig	Length contig	Coverage contig	*E*-value	Identity (%)	Mapped length (aa)	Percentage covered
Nitrate/nitrite	nark	kuste2335	405	485	3042	14.96	2.00E−144	86	393	97.0%
antiporter	nark	kuste2308	389	ND						
Nitrite transporter	focA	kusta0004	363	580	439	19.8	3.00E−73	86	145	39.9%
	focA	kusta0009	304	561	910	14.4	7.00E−25	100	69	66.7%
				571	405	20.2	5.00E−73	97	134	
	focA	Kuste3055	300	1289	1026	16.6	6.00E−117	80	245	81.7%
	focA	kustd1720	296	ND						
	focA	kustd1721	303	ND						
	focA	kuste4324	304	ND						
Nitrite reductase	nirS	kuste4136	621	ND						
NO forming	nirK	−	337	2818	556	11.67	7.00E−97	91	185	86.1%
				2821	445	10.5	5.00E−55	93	105	
Ammonium	amtB	kustc0381	585	2881	777	14	1.00E−99	84	228	78.8%
transport protein				2884	983	10.9	5.00E−69	67	233	
	amtB	kustc1015	448	ND						
	amtB	kuste3690	679	2219	3103	21.3	2.00E−119	53	476	70.1%
	amtB	kustc1012	451	306	1137	15.5	3.00E−119	77	282	98.9%
				283	1480	15.5	4.00E−68	72	167	
	amtB	kustc1009	519	272	735	16.2	3.00E−52	70	242	46.6%
Hydrazine	hao	kusta0043	591	523	680	13.6	6.00E−114	91	225	38.1%
or hydroxylamine	hao	kustc0458	554	1868	2043	18.9	0	89	507	91.5%
Oxidation	hzo	kustc0694	656	1210	1735	32.1	0	87	464	70.7%
	hzo	kustd1340	577	1210	1735	32.1	0	89	462	80.1%
	hao	kustc1061	536	2738	969	21	9.00E−167	82	321	59.9%
	hao	kuste2435	499	2239	746	14.9	7.00E−111	70	248	49.7%
	hao	kuste2457	433	ND						
	hao	kuste4574	584	2923	676	14.1	2.00E−131	87	347	59.4%
Hydrazine	hzsA	kuste2861	809	557	3360	40.5	2.00E−163	83	313	95.7%
synthesis				652	1594	32.8	0	81	461	
	hzsB	kuste2860	353	557	3360	40.5	0	84	346	98.0%
	hzsC	kuste2859	386	557	3360	40.5	8.00E−157	80	351	90.9%
Nitrate reductase	narG	kustd1700	1148	377	2006	30.7	0	86	584	97.4%
				409	1221	31.8	0	90	406	
			418	520	27	0	89	128	
	narH	kustd1703	410	434	1089	11.6	2.00E−142	70	334	81.5%
CO_2_ fixation	acsA	kustd1546	653	2544	667	16.2	6.00E−102	91	206	49.3%
				2530	747	11.6	8.00E−56	84	116	
	acsB	kustd1545	727	1103	466	11.9	8.00E−80	90	155	78.8%
				2517	878	12.1	8.00E−138	80	286	
				2529	476	5.6	5.00E−66	95	138	

### Phylogenetic analyses

**Figure 3:**
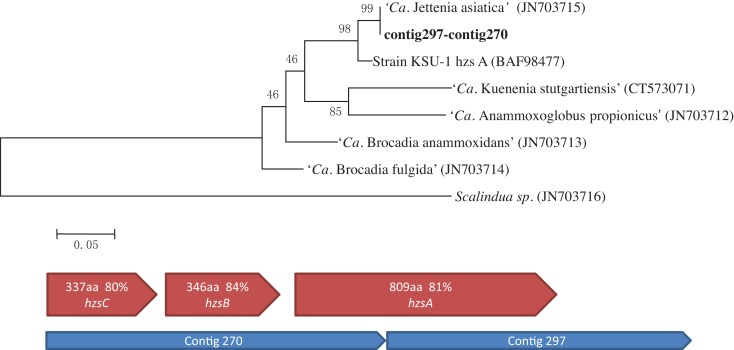
**Maximum likelihood trees indicating the phylogenetic relationships within the *Brocadiales* based on hydrazine synthase subunit A (hzsA) protein sequences**. Numbers at the nodes are percentages of bootstrap values based on 1000 re-samplings. NCBI accession number of reference sequences are shown in brackets (above). Similar organization of hydrazine synthase (hzsABC) genes cluster in *Jettenia* and *Kuenenia*. Blue pentagons represent *Jettenia* contigs contains HZS encoding genes, red pentagons indicating organization of Kuenenia hydrazine synthase gene cluster (below).

After the first assembly, the gene encoding the A subunit of HZS complex (hzsA) was recovered in two contigs (contig297–contig270). A Maximum likelihood tree of hzsA was then constructed using MEGA5 (Tamura et al., 2011), with reference sequences obtained from NCBI database (Figure 3).

Also, the 16S rRNA gene database was downloaded from the Ribosomal Database Project (RDP; release 10, update 29; Cole et al., 2009) and a subset of cultured or identified microorganisms (339,774 sequences) was used as a reference dataset. All reads generated were mapped to this dataset in order to investigate the composition of the other community members besides *Jettenia*. Read mapping was also performed on the genomes of anaerobic methane oxidizer “*Ca. Methylomirabilis. oxyfera*” (FP565575) and ammonium-oxidizing bacteria *Nitrosomonas eutropha* (NC_008344.1) and *Nitrosomonas europaea* (NC004757.1) to investigate the presences of other important community members.

## Results and Discussion

### Genes for anammox metabolism mapped by *Jettenia* contigs

The BLASTx results showed that most important anammox genes of *Kuenenia* could be identified in the assembled *Jettenia* contigs. The hit lengths of some of these with the target proteins, however, were relatively short (Table 1). A new assembly using reads mapped to KSU-1 genome increased the mapped length of some genes significantly. For example, for kustc0381, the mapped length increased to 78.8% of full length when using contigs from the new assembly (Table 2). However, for some other genes such as kustc0694, kuste1009, and kuste2308, the mapped lengths slightly decreased when using contigs generated from second assembly, or could not be mapped at all (Table 2).

More sequence data with longer read length might lead to a more efficient assembly and would subsequently resolve this problem, but at this moment, we believe that the data presented here using the combination of contigs from two assemblies was sufficient to provide evidence that the predicted genes exist in the *Jettenia* genome.

### Transportation of nitrite/nitrate and ammonium

All anammox bacteria have to take up inorganic nitrogen substrates (ammonium, nitrite, and nitrate) from the environment, where they usually exist in limiting amounts. Furthermore, under oxygen-limited conditions, anammox bacteria may have to compete for ammonium with other community members such as heterotrophs and aerobic ammonium-oxidizing archaea (AOA) or bacteria (AOB). Under these conditions, there will also be competition for nitrite with nitrite-oxidizing bacteria (NOB; Fussel et al., 2012). Therefore, many genes encoding for nitrite, nitrate, and ammonium transport were present in the genomes of anammox bacteria most notably ammonium-transporter *amtB*, nitrate/nitrite antiporter *narK*, and formate/nitrite transporter *focA*.

Five copies of *amtB* were identified in the genome of *Kuenenia* (Strous et al., 2006). In the *Jettenia* metagenome, seven contigs were found to have best BLASTx hit with these five *amtB* proteins with an average identity of 66% (Table 1) on amino acid basis, which indicated that all five *amtB* genes were very likely also encoded by the genome of *Jettenia*. Six *focA* genes were found in *Kuenenia* genome, but only three of them (kusta0004, kusta0009, and kuste3055) could be partially retrieved from the metagenome of *Jettenia* (Tables 1 and 2). In the genome of *Brocadia* also only one gene encoding for *focA* protein could be identified which shared a 79% similarity with kusta0004 (Gori et al., 2011). However, in the genomes of *Scalindua* and KSU-1 multiple genes encoding *focA* were indentified (Van de Vossenberg et al., 2012). This might reflect the ability of this this marine anammox to thrive in nutrient limited conditions, such as marine oxygen-minimum zones (OMZ) where the concentration of inorganic nitrogen compounds are usually very low (Lam and Kuypers, 2011). On the other hand, less *focA* protein encoding genes in *Brocadia* and *Jettenia* genomes might indicate that those species possess other proteins which also function as nitrite transporter. In the genome of KSU-1, however, five formate/nitrite transport Two of the genes found in KSU-1 (ZP_10099012 and ZP_10100997), shared 82% identity and have best BLASTp hit with kuste3055. The other three (ZP_10098545, ZP_10098546, ZP_10098796) have best hits with kusta0009, kusta0004, and kustd1720, respectively.

For nitrate transport, two gene copies encoding a putative nitrate/nitrite antiporter *narK* (kuste2308, kuste2335) in the *Kuenenia* genome were partially mapped on *Jettenia* contigs (Tables 1 and 2).

### Nitrite/nitrate conversion

Once transported, inorganic nitrogen substrates must be converted in several steps in the anammox metabolism, resulting in dinitrogen gas. First, nitrite is reduced to NO, this reaction could be catalyzed by a cytochrome *cd_1_*-type nitrite reductase *nirS* in *Kuenenia* (Strous et al., 2006). The *nirS* encoding genes were also identified the metagenome of *Scalindua* (Van de Vossenberg et al., 2012). The expression level of those genes analysed by transcriptomic and proteomic studies, however, were different: The *Scalindua* transcriptome data showed that *nirS* was highly expressed (Van de Vossenberg et al., 2012), but the *nir*S expression levels in *Kuenenia* were much lower (Kartal et al., 2011). In our *Jettenia* assembly, the *nirS* of *Kuenenia* (kuste4136) could not be mapped by any contig. BLASTx search also showed that none of the contigs has best hit with this gene. *NirS* also seemed to be absent from the metagenome of *Brocadia fulgida* (Gori et al., 2011). Again, the low sequencing depth could be an explanation for the absence of *nirS* gene. However, since at least one copy of all other important genes for anammox metabolism could be (partially) retrieved, the absence of *nirS* suggested that *Jettenia* was likely to employ another way to mediate the conversion of nitrite to NO. Very recently, the genome analysis of anammox strain KSU-1 showed that instead of *nirS*, a copper nitrite reductase *nirK*-homolog was detected. Biochemical studies confirmed that this nirK catalyzed the reduction of nitrite to NO (Hira et al., 2012). The *nirK* gene encodes a copper-containing nitrite reductase (CuNIR), which was previously identified in denitrifiers, but had not been detected in anammox bacteria. After mapping our *Jettenia* contigs to the KSU-1 assembled contigs, two *Jettenia* contigs (contig1180 and contig21694) covered almost the full length of *nirK* with only a gap of 21 amino acids. Together with the BLASTx search result (Tables 1 and 2), this strongly suggested that *Jettenia* also contained a *nirK* gene as an alternative for *nirS* to convert nitrite to NO.

To reduce nitrate to nitrite, a nitrate reductase complex is required, including *narG* and *narH*. In the *Kuenenia* genome, both genes were retrieved and together with other three genes formed a large gene cluster encoding a nitrate reductase complex (Strous et al., 2006). Large parts of *Kuenenia narG* and *narH* could be mapped by *Jettenia* contigs, which indicated both genes were present in the genome. In anammox bacteria, however, it is believed that *narGH* normally functions in the reverse way as a nitrite oxidoreductase nxrAB and would catalyze the oxidation of nitrite to nitrate (Jetten et al., 2009). Therefore, the *narGH* retrieved from Jettenia genome could also function in this way, similar to other anammox bacteria.

### Hydrazine metabolism and N_2_ formation

NO, produced thorough nitrite reduction, and ammonium are are the direct precursors for the production of hydrazine (N_2_H_4_). HZS, encoded by gene cluster kuste2859-2861 (*hzsCBA*) in the *Kuenenia* genome (Kartal et al., 2011), catalyzes this reaction. The genes of the HZS cluster (kuste2859-2861) were the most highly expressed genes in both proteome and transcriptome data of *Kuenenia* (Kartal et al., 2011). In the transcriptomic and proteomic analyses of *Scalindua*, this was also observed (Van de Vossenberg et al., 2012). In the *Brocadia* metagenome, the coverage of contigs containing *hzs* genes was much higher than other contigs, which might be the result of multiple copies (Gori et al., 2011). Also here, this gene cluster could be perfectly mapped by two *Jettenia* contigs (contig297 and contig270) with identities between 80 and 84% (Table 1; Figure 3). Based on the BLASTx search, the two contigs should be concatenated together, but the metagenomic assembly failed to link them probably as a result of low coverage at the linking region. Moreover, the coverages of contig297 and 270 were 52 and 30-fold, respectively. This was much higher than the average coverage of contigs listed in Table 1, which was consistent with findings in other anammox genome assemblies (Gori et al., 2011).

Finally, an octaheme hydroxylamine oxidoreductase-like (*hao*-like) protein, HDH, catalyzes the oxidation of hydrazine to produce dinitrogen gas (Kartal et al., 2011). In the *Kuenenia* genome, at least 8 *hao*-like octaheme proteins were identified (Strous et al., 2006). Two of them, kustc0694, and kustd1340 were predicted to function as true HDHs. Both of them could be mapped by one same *Jettenia* contig, the contig8275 with high identities (Table 1), which is not surprising because the amino acid sequences of kustc0694 and kustd1340 are 96.4% identical.

Other *hao*-like proteins retrieved from *Kuenenia* genome could also be partially mapped by assembled *Jettenia* contigs, some of them, such as kustc0458, were nearly fully mapped (Tables 1 and 2). These *hao*-like proteins might be involved in some other reactions in the anammox metabolism, such as electron transfer, detoxification of potentially hazardous nitrogen compounds, dissimilatory nitrate/nitrite reduction to ammonia (DNRA) or ammonia formation (Campbell et al., 2009; Kartal et al., 2011; Van de Vossenberg et al., 2012).

### Carbon fixation

Previous studies indicated that the carbon fixation by anammox bacteria was accomplished through the acetyl-coenzyme A (CoA) pathway (Schouten et al., 2004), with reducing power required supplied by hydrazine oxidization (Kartal et al., 2011). A complete acetyl-CoA pathway was identified in the genome of *Kuenenia* (Strous et al., 2006). Two genes encoding the key enzyme in this pathway, the carbon monoxide dehydrogenase/acetyl-CoA synthase subunit alpha and beta (*acsA* and *acsB*) were retrieved with high identities to genes of *Kuenenia* in seven and five contigs by both assemblies, respectively (Tables 1 and 2), indicating *Jettenia* employed the same carbon fixation pathway as the previously studied anammox bacteria.

### Other important community members

Previous phylogenetic analyses of the same granule sample indicated a highly diverse community. Aerobic ammonia-oxidizing bacteria (AOB) were detected with a low abundance (Quan et al., 2008). In addition, the nutritional condition in the granule reactor would also allow the growth of other microorganisms such as methanogens and methane oxidizers or the recently reported denitrifying methanotroph “*Ca*. Methylomirabilis oxyfera” (Ettwig et al., 2010). The MetaCluster grouping result suggested that two thirds of the 37,432 assembled contigs were not originating from *Jettenia* but from other community members. Moreover, only ∼7% reads could be mapped to the KSU-1 genome. Therefore, it was likely that there were other members in the community with significant abundances. Analyses based on 16S rRNA genes and read mapping to reference genomes were performed to investigate the community diversity.

Mapping all sequence reads to a subset of 339,774 16S rRNA gene sequences of the RDP database confirmed a high abundance of *Jettenia*, with 15.6-fold coverage. A clone from a methanogenic archaeon (JN836400) was the second most abundant, with 4.6-fold coverage. Other abundant community members belonged to the *Burkholderia* (HQ222273) with 2.6-fold coverage and the *Chloroflexi* (HQ133206) with 2.1-fold coverage.

Since a methanogen was the second most abundant organism as detectable by 16S mapping, we decided to investigate this group of organisms in more detail. A *mcrA* gene, encoding the methyl coenzyme M reductase alpha subunit and a phylogenetic marker for methanogens, was detected on contig 9376 with a best hit to *Methanothermobacter thermoautotrophicus* (91% identity at amino acid level). Further, all contigs in group 2 were submitted to KEGG Automatic Annotation Server (KAAS; Moriya et al., 2007) for pathway mapping. A nearly complete methanogenesis pathway was retrieved with 82 contigs involved.

Since the granular sludge was originated from an anaerobic reactor system with ample supply of nitrite in the influent, the presence of methane could create a suitable niche for the anaerobic methanotroph “*Ca*. M. oxyfera”. Indeed mapping the reads to the *“Ca*. M. oxyfera” genome resulted in a mapping of 131,689 reads. Based on the amount of reads that could be mapped to KSU-1 (1,060,817), this resulted in an estimate of ∼6% abundance of “*Ca*. M. oxyfera” in the enrichment culture. Based on these findings we hypothesize that a full methane cycle is present in the enrichment.

**Figure 4:**
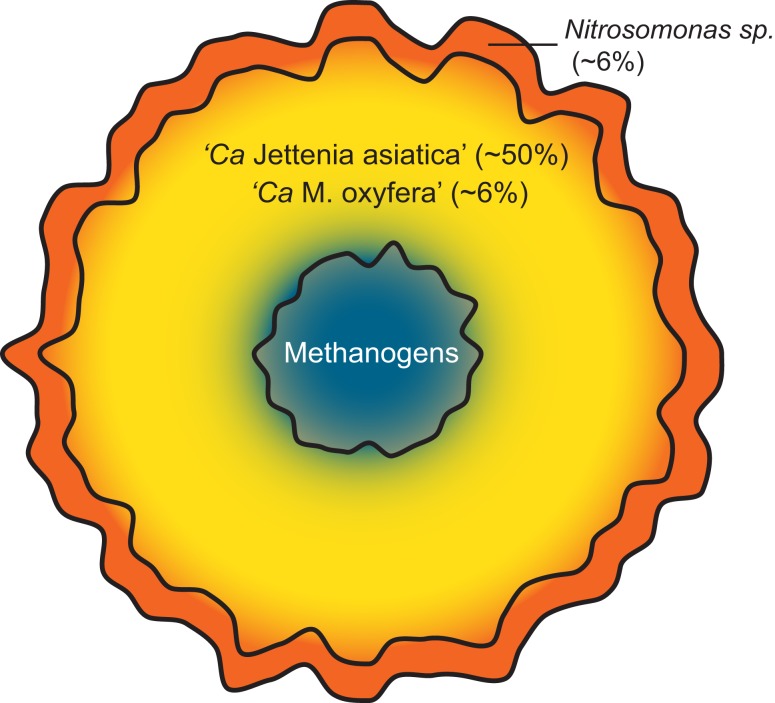
**Schematic model of the community composition of the sludge granules**. The microaerobic conditions in the outer layer of the granule (red) allow *Nitrosomonas* sp. to convert ammonium to nitrite. In the completely anaerobic interior of the granule (blue) methanogens form the last step in the anaerobic degradation of organic matter and produce methane. The methane formed allows coexistence of NC10 bacterium *“Ca*. Methylomirabilis oxyfera”, which oxidizes methane using nitrite as electron acceptor, with the dominant anammox bacterium *“Ca*. Jettenia asiatica” (yellow). Abundance of “*Ca*. Jettenia asiatica” is estimated based on qPCR (Quan et al., 2008). Abundance estimates of *“Ca*. Methylomirabilis oxyfera” and *Nitrosomonas sp*. are based on read mapping on the genomes of “Ca. Methylomirabilis oxyfera”, *Nitrosomonas europaea* and *Nitrosomonas eutropha*. No abundance estimate could be given for the methanogen community, since the *Jettenia* reference abundance was taken with Bacteria specific primers.

As *Nitrosomonas sp*. were previously detected (Quan et al., 2008), we also performed a read mapping on the two available *Nitrosomonas* genomes; *N*. *europaea* and *N. eutropha*. 90,095 and 30,150 reads could be mapped to these genomes respectively, leading to an abundance estimate of ∼6% *Nitrosomonas* sp. in the enrichment. These findings suggest that a small oxygen gradient is present in the granular sludge, increasing the amount of nitrite available for *Jettenia* (Figure 4).

There were still more than 50% reads which could neither be mapped to any references mentioned above, nor be assembled into contigs. Additionally, less than 7% of the reads could be mapped to the genome of KSU-1. Since this organism is closely related to *Jettenia*, this leads to an abundance estimate that is much lower than previous reported (50%). On the other hand, 16S rRNA gene mapping confirmed the earlier qPCR analysis which indicated that *Jettenia* is the dominant bacterium in the enrichment. However, the 50% abundance of anammox bacteria was based on the 16S rRNA gene analyses with bacteria specific primers (Quan et al., 2008). Archaea, such as methanogens, were not included in the calculation and neither were Eukaryotes. However, Archaea were included in the 16S mapping and did not constitute a larger fraction than Jettenia. Thereby, Eukaryotes could affect the sequencing results significantly even when present at low abundance, because of their relatively large genomes. Therefore, we hypothesize that the majority of the reads obtained from the enrichment originated from a low abundance Eukaryotic population.

## Concluding Remarks

Here we presented sequencing and metagenomic analysis of a community enriched for anammox bacterium “*Candidatus* Jettenia asiatica.” Although it was impossible to distil a high quality draft genome of this organism from the metagenome, we were able to identify all essential genes for anammox metabolism based on the comparison with other anammox bacteria such as *Kuenenia* and KSU-1. The identification of a copper-containing nitrite reductase NirK in *Jettenia* genome instead of cytochrome *cd*_1_-type nitrite reductase NirS indicated a flexible metabolism of anammox bacteria in terms of nitrite reduction to NO. However, the role of this enzyme, as well as the evolutionary relationship between *Jettenia* and other anammox bacteria still need to be studied.

Additionally, we could confirm earlier results on the presence of methanogens as important players in the side population and have shown clear indications of the presence of anaerobic methanotroph *“Ca*. M. oxyfera” in the culture. Together with anammox bacteria and aerobic ammonium oxidizers, these microorganisms formed a mutualistic community (Figure 4). Oxygen cannot penetrate into the very core of the granule, which could allow the growth of methanogens. The methanogens produce methane which is required for the anaerobic methanotroph. However, such a theory still needs further studies to confirm.

## Conflict of Interest Statement

The authors declare that the research was conducted in the absence of any commercial or financial relationships that could be construed as a potential conflict of interest.
